# Polypropylene Hollow-Fiber Membrane Made Using the Dissolution-Induced Pores Method

**DOI:** 10.3390/membranes12040384

**Published:** 2022-03-31

**Authors:** Zhongyong Qiu, Chunju He

**Affiliations:** The State Key Laboratory for Modification of Chemical Fibers and Polymer Materials, College of Materials Science and Engineering, Donghua University, Shanghai 201620, China; chunjuhe@dhu.edu.cn

**Keywords:** PP membrane, dissolution-induced pore method, polypropylene hollow-fiber membrane

## Abstract

The efficient preparation of hydrophilic polypropylene membranes has always been a problem. Here, a twin-screw extruder was used to melt-blend ethylene-vinyl alcohol copolymer and polypropylene; then, hollow fibers were extrusion-molded with a spinneret and taken by a winder; after this, dimethyl sulfoxide was used to dissolve the ethylene-vinyl alcohol copolymer of the fiber to obtain a polypropylene hollow-fiber membrane. This procedure was used to study the effects of different contents and segment structure of ethylene-vinyl alcohol copolymer on the structure and filtration performance of the membranes; furthermore, the embedded factor and blocked factor were used to evaluate the ethylene-vinyl alcohol copolymer embedded in the matrix without dissolving and or being completely blocked in the matrix, respectively. The results show that the increase in ethylene-vinyl alcohol copolymer could reduce the embedded factor and increase the blocked factor. The increase in the polyethylene segments of ethylene-vinyl alcohol copolymer could increase both the embedded factor and blocked factor. The water permeation of the membrane reached 1300 Lm^−2^·h^−1^·bar^−1^ with a 100% rejection of ink (141 nm) and the elongation at break reached 188%, while the strength reached 22 MPa. The dissolution-induced pores method provides a completely viable alternative route for the preparation of polypropylene membranes.

## 1. Introduction

Polypropylene hollow-fiber membrane (PPHFM) is widely used in water treatment [1,2,3], industrial purification [4,5], and pharmaceutical separation [6,7,8]. Currently, polypropylene (PP) hollow-fiber membranes are mainly prepared with the thermally induced phase separation method (TIPS) and the melt-stretching method (MS) [9,10]. The studies on these two methods are comprehensive. For example, the phase separation process [11,12,13,14,15], diluents [7,8,10,16,17], solidification bath [11,12,13,14], and kinetic thermodynamics [18,19] in the TIPS have been reported on in detail. Similarly, research on co-blending grafting [20,21,22,23,24,25,26], stretching [27,28,29], annealing [30,31], and crystallization [5,9,30,32] in the MS has been conducted.

However, the limitation of the TIPS and MS [10,17,19,33] is that the PP molecule has no polar groups, so it is difficult for it to form effective secondary bonds with water molecules; therefore, the poor hydrophilicity makes the pores clog easily, resulting in a lower permeation and poor durability [11,34,35,36,37,38]. These effects lead to the need to perform complex post-processes on the PPHFM, such as grafting [11,35,39,40] and coating [37,38,41,42]. The dissolution-induced pores method (DIP), a neglected technology used for the preparation of PPHFMs, is not discussed in the available research papers. The DIP differs from the TIPS and MS in terms of its technical principles: it is not only simple enough to control the microstructure of the membrane, but also uncomplicated enough to combine hydrophilic modification and pore formation into one step.

In this study, the DIP method was adapted to melt-blended and extruded ethylene-vinyl alcohol copolymer (EVOH) functioning as a dissolvable part with PP; it was used to dissolve the EVOH with dimethyl sulfoxide (DMSO) to obtain hydrophilic polypropylene hollow-fiber membranes. The filtration performance of the hollow-fiber membranes was investigated with different EVOH contents, and this procedure was carried out to investigate the effect of different types of EVOH on membrane performance under optimal EVOH content conditions. The embedded factor was used to quantitatively evaluate the EVOH that was embedded in the matrix without being dissolved, and the blocked factor was used to quantitatively evaluate the EVOH that was completely blocked in the PP. Our groundbreaking research should provide a foundation for the development of the DIP method.

## 2. Materials and Methods

### 2.1. Materials and Equipment

The ethylene vinyl alcohol copolymer (EVOH, Nippon Synthetic Chemical Industry Co., Osaka, Japan) with ethylene segments of 24%, 27%, 29%, 32%, 38%, and 44% was dried under vacuum with an oven at 40 °C for 12 hours. Polypropylene was purchased from Sinopec Co., Ltd. (Beijing, China), with a melt index of 3.5 g/10 min. Dimethyl sulfoxide (DMSO, 99.9%) was used after de-watering by molecular sieve. The ink (Shanghai Hero Co., Ltd., Shanghai, China) was used to evaluate the rejection of membranes. A twin-screw extruder (TS-18) with a screw diameter of 1.8 cm and a length of 150 cm was supplied by Nanjing Huaju Co., Ltd. (Nanjing, China); the inner diameter and outer diameter of the spinneret were 1.2 mm and 1.8 mm, respectively.

### 2.2. Preparation of Hollow-Fiber Membranes

The EVOH (24% ethylene) and PP were mixed with a twin-screw extruder, with a ratio of EVOH from 38 wt.% to 48 wt.%. The temperature of the zones (TS-18) was 90 °C, 160 °C, 180 °C, 180 °C, and 180 °C, respectively; the host speed was 130 r/min, the winding speed was 1.2 m/s, and the stretching ratio was 6 times. The hollow fibers were cut into suitable lengths and soaked with DMSO for 24 hours; they were taken out to dry for testing and named m38 to m48 based on their content of EVOH.

The EVOH and PP were mixed with the mass of EVOH, which was 42 wt.%, wherein the ethylene content of EVOH was 24%, 27%, 29%, 32%, 38%, and 44%. The spinning-related process parameters were the same as described above, and the hollow-fiber membranes were named from M24 to M44 according to their content of ethylene.

### 2.3. FTIR, Particle Size/Zeta Potential, Pore Size Testing, and SEM Testing

The Fourier Transform Infrared Spectrometer (FTIR, Nicolet iS50, Madison, WI, USA) was used to obtain the molecular structure information. A nanoparticle size and zeta potential analyzer (Anton Paar, Graz, Austria) was used to evaluate the size of ink nanoparticles (Shanghai Hero Co., Ltd.). The samples of PPHFM were freeze-dried, and a scanning electron microscope (SEM SU8010 Hitachi, Minato-ku Tokyo, 1.5 kV, 10 μA) was used. The pore size distribution was tested by a liquid–liquid pore size analyzer (PSMA-10, Nanjing, China).

### 2.4. Embedded and Blocked Factor

The experiments were repeated 3 to 5 times, and the errors were displayed in the form of error bars.

The embedded factor is defined as the mass ratio of the substance that is not dissolved for encapsulation by matrix. The blocked factor is defined as the mass ratio of the substance that is not dissolved due to its compatibility blend with the matrix.

For example, the m40’s embedded factor and blocked factor are calculated as follows:

The mass of m40 before being soaked by DMSO is *m*_1_ (g), and the theoretical EVOH content is *m*_0_ (*m*_0_ = *m*_1_ × 40%); the mass of m40 is *m*_2_ (g). The *m*_3_ (g) is obtained after the m40 is crushed with liquid nitrogen and then washed by DMSO and dried to obtain the mass. 

The embedded factor (*p*) is calculated by:

(1) $$p=\frac{m_{2}-m_{3}}{m_{0}}\times 100\%$$

The blocked factor (*b*) is calculated as follows:

(2) $$b=\frac{m_{0}-\left(m_{1}-m_{3}\right)}{m_{0}}\times 100\%$$

### 2.5. Filtration Performance and Porosity

**Figure 1:**
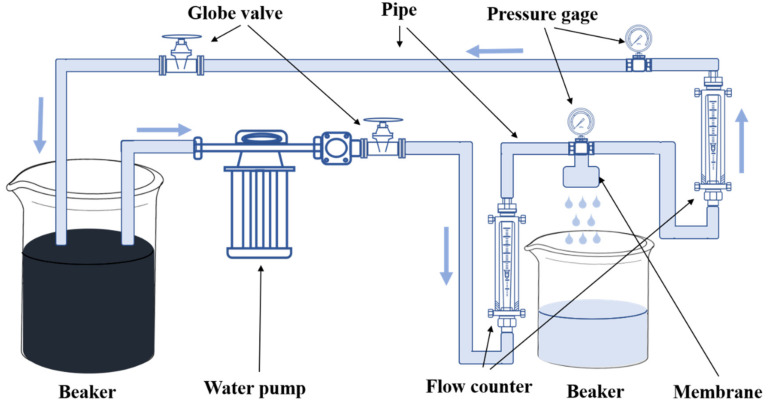
Schematic diagram of the hollow-fiber membrane permeation and rejection test device.

The permeation of the membranes was tested with the device developed by our group (Figure 1), and calculated as follows:

(3) $$F=\frac{V_{f}}{tPS}$$

where *F* (Lm^−2^·h^−1^·bar^−1^) is the permeation, *V_f_* (L) is the volume of filtrated water, *P* (0.1 MPa) is the pressure, *S* (m^2^) is the area of filtration, and *t* (h) is the filtration time.

The rejection of ink was determined by a UV absorption spectrometer (Shimadzu 1800, λ = 307nm) via measuring absorbance with the standard curve method [43], which was calculated as follows:

(4) $$R=\frac{A-A_{0}}{A}\times 100\%$$

where *R* (%) is the rejection, *A* is the absorbance of the unfiltered liquid, and *A*_0_ is the absorbance of the filtered liquid.

The porosity of membranes was tested with wet method [44,45] and calculated by:

(5) $$Pr=\frac{m_{4}-m_{5}}{\rho_{water}V_{0}}\times 100\%$$

where *Pr* (%) is the porosity, *V*_0_ (cm^3^) is the membrane volume, *m*_4_ (g) is the wet membrane mass, *m*_5_ (g) is the dry membrane mass, and *ρ_water_* (1 g/cm^3^) is the water density. 

### 2.6. Mechanical Test

The elongation at break was tested by a tensile machine (Shenzhen Kaiqiang Co., Ltd., Shenzhen, China) and calculated by:

(6) $$Er=\frac{∆L}{L_{0}}\times 100\%$$

where *Er* (%) is the elongation at break, Δ*L* (%) is the change in sample in length at break, and *L*_0_ (%) is the length of the sample before testing.

The strength was calculated as follows:

(7) $$T=\frac{f}{S_{0}}$$

where *T* (MPa) is the strength, *f* (N) is the force, and *S*_0_ (m^2^) is the cross-sectional area of the sample. 

## 3. Results and Discussion

### 3.1. Preparation of Hollow-Fiber Membranes (with Different Contents of EVOH) 

**Figure 2:**
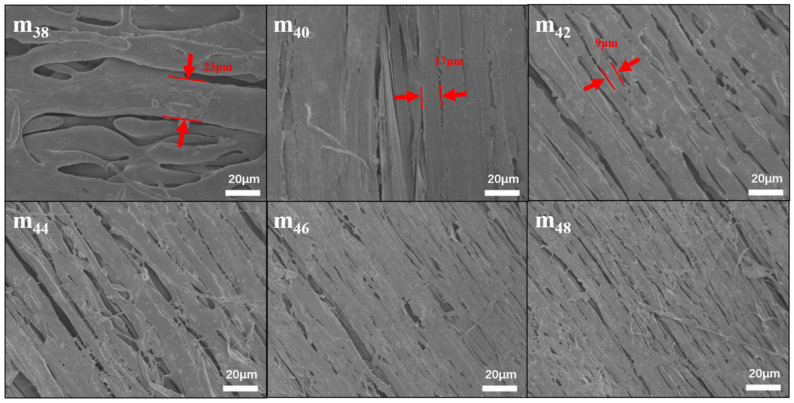
Surface morphology of hollow-fiber membranes with different contents of EVOH (24% ethylene).

Figure 2 shows the surfaces of the PPHFMs prepared by the DIP method with EVOH. The PPHFMs show a unique structure of microfibers, and the diameter of the microfibers decreases as the EVOH content increases. Polymers’ incompatibility is the main reason for the microfibrillation of blends after stretching [46,47,48], so it is possible that EVOH and PP are not fully compatible during the blending process; the shearing cuts the sizes of the PP and EVOH into small phases, and then the stretching process promotes the microfibrillation of PP. So, the increase in EVOH content promotes the progress of microfibrillation, making the microfibrils of PP more obvious, which suggests that the content of EVOH can directly control the surface of the PPHFM and greatly reduce the difficulty of controlling microfibrillation.

**Figure 3:**
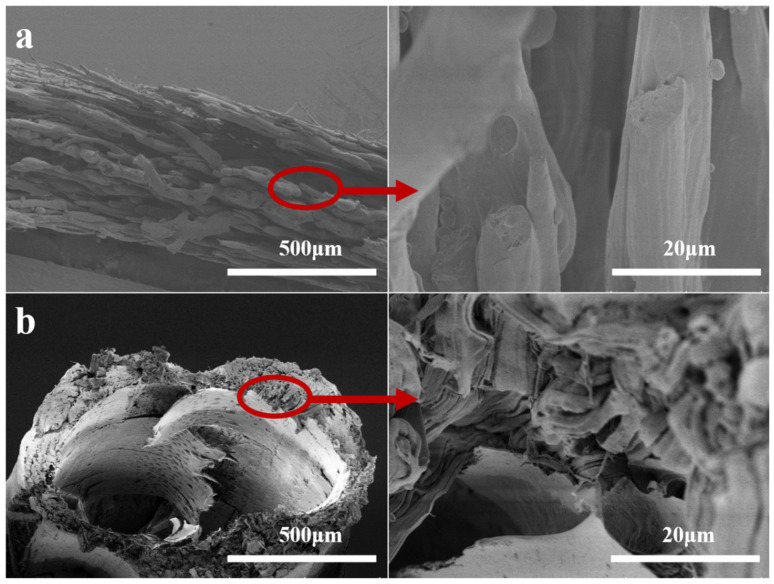
Cross morphology of hollow-fiber membranes m38 (**a**), m48 (**b**).

Figure 3 shows the cross-sectional morphologies of the PPHFMs when the EVOH content is 38 wt.% and 48 wt.%, respectively. Compared with the surface topography of the PPHFMs (Figure 2), the cross-section of the m38 is similar to its surface, both of which are superimposed and compacted by microfibers with branches, but the microfibers in the surface topography show a certain orientation: the cross-section microfibers appear to be very chaotic. The m40 is also similar in terms of its surface morphology; the microfibrillation of the section is obvious. Different from the microfibers of the m38 section, the microfibers of the m40 section are more slender, and their orientation is more obvious, which is similar to the surface morphology of the m40. 

**Figure 4:**
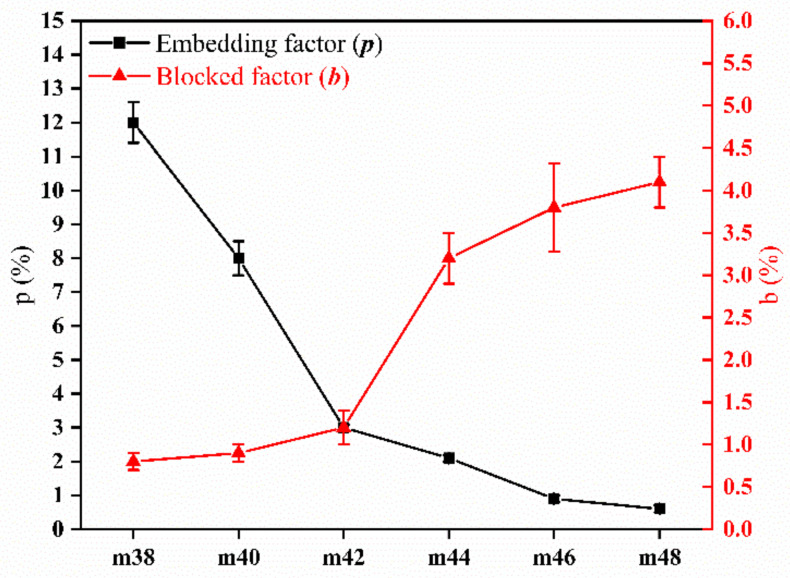
Blocked factor *p* and embedded factor *b* of hollow-fiber membrane with different EVOH (ethylene chain segment content of 24%) contents.

Figure 4 shows the changes in the blocked and embedded factor of EVOH in the PPHFM with different contents of EVOH. The pore-forming process of the membrane prepared by the dissolution method is also the process of the polymer being dissolved. However, it cannot be ensured that all the soluble polymers can be removed, and the soluble polymers that cannot be removed can be divided into two cases: one is that the EVOH should be dissolved, but due to the complete encapsulation of the PP, it cannot be exposed to the DMSO and cannot be removed; the other is that the soluble EVOH and the PP are continuously sheared to achieve complete miscibility. The EVOH, in this case, is also difficult to remove. In order to distinguish the above two cases, the blocked coefficient (*b*) and embedding coefficient (*p*) were defined to quantify the description. With the increase in EVOH content, the embedded factor continued to decrease, and the blocked factor continued to increase (Figure 3). When the EVOH content increased from 38 wt.% to 48 wt.%, the embedding factor decreased from 12% to 0.6%, whereas the blocked factor increased from 0.8% to 4.1%, an increase of more than five times. This shows that, in the process of increasing the EVOH content, the embedded EVOH gradually decreased, but the EVOH blocked in the PP gradually increased. 

**Figure 5:**
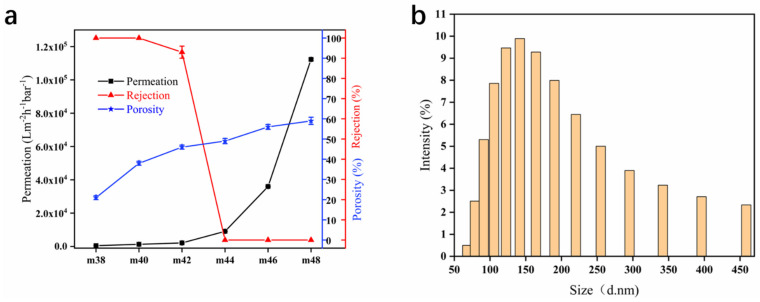
Changes in permeation, rejection and porosity of hollow-fiber membranes at different EVOH (ethylene chain segment content of 24%) contents (**a**) and the size and distribution of ink particles (**b**).

Figure 5 shows the filtration performance and porosity of the hollow-fiber membranes with different EVOH contents and the size of the particles in the ink. In general, with the increase in EVOH content, the rejection of the PPHFM showed a decreasing trend, whereas the permeation and porosity showed an increasing trend. When the EVOH content is 40 wt.%, the hollow-fiber membrane’s rejection of the ink reaches 100% and the water permeation is 1300 Lm^−2^·h^−1^·bar^−1^. When the EVOH content exceeds 40 wt.%, the rejection drops rapidly and the permeation rises sharply. Compared with the surface topography of the PPHFM (Figure 2), although the uniformity of the microfiber increased with the increasing EVOH content during the microfibrillation process, such topographic changes did not contribute to the rejection. The contribution of microfibrillation is an increase in permeation when the EVOH content increases, but when the EVOH content exceeds 44 wt.% the rejection drops to 0%, resulting in membrane failure. The above data also illustrate that EVOH content is the main factor controlling the membrane filtration performance. The ink is an aqueous solution of carbon particles that is used to detect the rejection performance of the membrane, and its particle size distribution is obviously concentrated (Figure 5b); the most probable particle size distribution is 141 nm.

**Figure 6:**
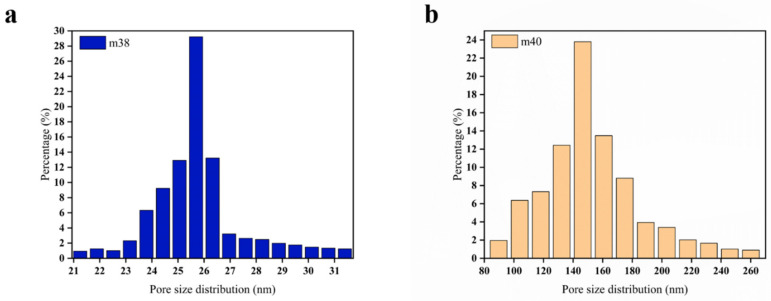
Pore size distribution of hollow-fiber membranes m38 (**a**) and m40 (**b**).

Figure 6 shows the pore size distribution test results of the hollow-fiber membranes with an EVOH content of 38 wt.% and 40 wt.%. In the permeation and rejection tests, the ink rejection of the membranes m38 and m40 was greater than 90% (Figure 5), so these two membranes were chosen for pore size analysis for comparison and discussion. It can be seen from the results in Figure 6 that the pore size distribution range of m38 is 22–30 nm, whereas that of m40 is 80–210 nm. Obviously, compared with m40, the pore size distribution of m38 is narrower, and the membrane pore size is smaller. This shows that in the DIP method, increasing the EVOH content can expand the pores but also make the pore size distribution wider.

**Figure 7:**
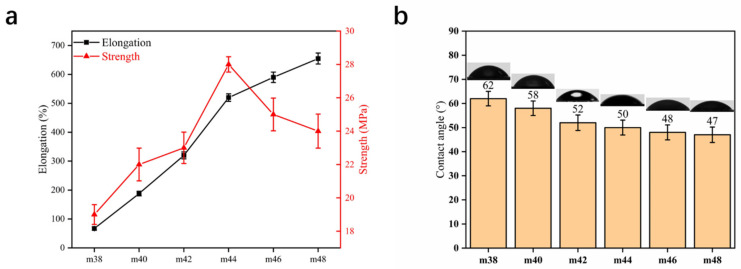
Mechanical properties (**a**) and contact angle (**b**) of PPHFM with different EVOH (ethylene chain segment content of 20%) contents.

Figure 7 shows the mechanical properties and pure water contact angle of the hollow-fiber membranes. Good mechanical properties guarantee the membranes’ service. The elongation of the membrane increases from 67% to 655% when the EVOH content increases from 38 wt.% to 48 wt.%. The strength of the PPHFM shows a different tendency; it rises from 19 MPa to 28 MPa and then falls to 24 MPa. This interesting phenomenon may come from the two changes brought about by microfibrillation: thinner fibers result in an increased elongation at break, and the thinner fibers also reduce the strength as a result of fewer microfibers sticking to each other (Figure 2).The reduction in microfiber contact points leads to an increase in the independence of microfibers during the stretching process; the lack of an overall connection and the stress caused by structural changes is transmitted to all microfibers, so the strength decreases. When the content of the EVOH is 40 wt.%, the elongation at break is 188% and the strength reached is 22 MPa. The water contact angle decreases with the increasing EVOH content, which also confirms the trend of the blocked factor *b*; the hydrophilicity of the PPHFM can only be inherited from the EVOH because PP is a hydrophobic material [40]. Thus, the blocked mass of the EVOH in the PP was reflected in the change in the water contact angle.

### 3.2. Preparation of Hollow-Fiber Membranes (EVOH with Different Ethylene Segments)

**Figure 8:**
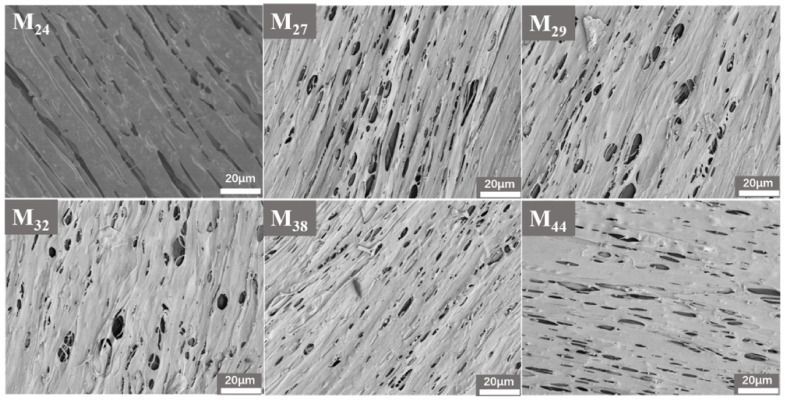
Surface morphology of EVOH hollow-fiber membranes with different ethylene chain segment contents.

The DIP method should be further investigated for the intrinsic law. Thus, the EVOH (with a mass ratio of 42 wt.%) was studied with different ethylene segment contents. Figure 8 shows that the microfibrillar structure was weakened by increasing the cross-linking of points with each other as the ethylene segment increased, and the pores were changed from extremely narrow to elliptical, which was related to the increase in the ethylene segments of the EVOH, leading to improved compatibility with PP and less dissolution of the EVOH. 

**Figure 9:**
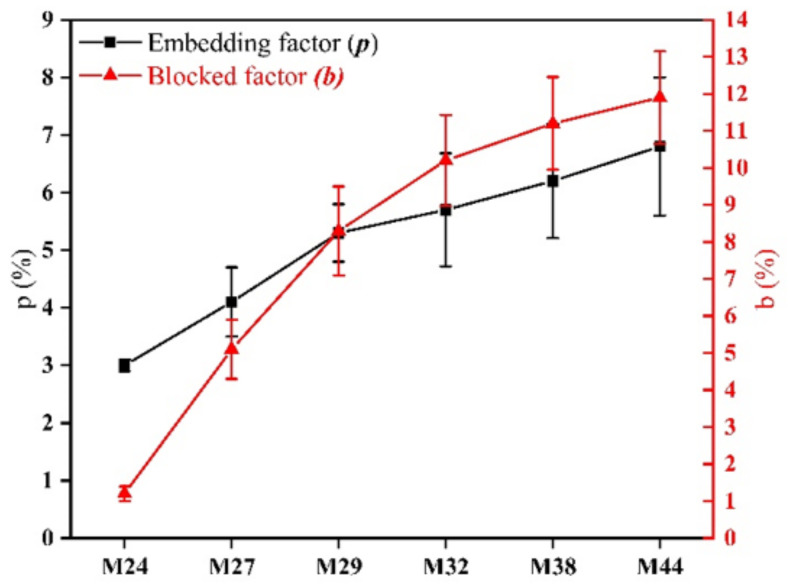
Blocked factor b and embedded factor p of hollow-fiber membranes (EVOH with different ethylene chain segment contents).

Figure 9 shows the blocked factor and embedded factor of PPHFM with the different kinds of EVOH. It is clear that the embedding and blocking factors increased with the increase in the polyethylene chain segments of the EVOH. The embedded factor was increased from 3% to 6.8% and the blocking factor was increased from 1.2% to 11.9%. This indicates that the compatibility with the PP matrix is enhanced with the increase in the polyethylene segments of the EVOH, which is also reflected in the disappearance of microfibrils on the membrane surface (Figure 8).

**Figure 10:**
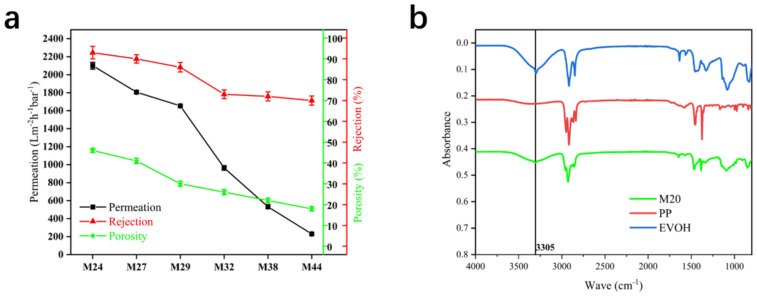
(**a**) Permeation, rejection, and porosity of EVOH hollow-fiber membranes with different ethylene chain segment contents, (**b**) FTIR of membrane M24, PP, and EVOH.

Figure 10a clearly illustrates that the permeation, rejection, and porosity of the membrane decreased with the increase in the EVOH’s polyethylene section. The permeation of the membrane decreased from 2100 Lm^−2^·h^−1^·bar^−1^ to 230 Lm^−2^·h^−1^·bar^−1^, the rejection decreased from 93% to 70%, and the porosity decreased from 46% to 18%. Because the porosity decreased, the water flow was directly reduced, but at the same time as the porosity decreased, the rejection also decreased, indicating that the pore size of the membrane was increasing, as can be directly observed in Figure 8. Such changes may be due to an increase in the embedded factor (Figure 9), which reduces the porosity and increases the pore size. Thus, it is not a good choice to increase the polyethylene segment in the EVOH to improve the performance of the membrane. However, it is helpful for us to better understand the mechanism of the DIP. The increase in the blocked factor indicates that the amount of undissolved EVOH increased, which explains the decrease in porosity, and the FTIR (3305 cm^−1^, stretching vibration of −OH) of M24 verified that some EVOH remained on the membrane (Figure 10b).

**Figure 11:**
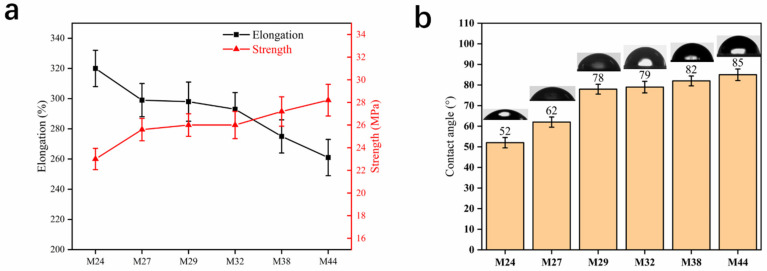
Mechanical properties (**a**) and water contact angle (**b**) of EVOH hollow-fiber membranes with different ethylene chain segment contents.

Figure 11 shows the mechanical properties and water contact angle of the membranes. The elongation of the membranes exhibited a gradual decreasing trend from 320% to 261%, but the strength exhibited a gradual increasing trend from 23 MPa to 28.2 MPa. Changes in mechanical properties are often related to changes in structure, and it is reasonable to conclude that the reduction in microfibers increases the strength of the membrane and reduces the elongation. Thus, it is feasible to adjust the mechanical properties of hollow-fiber membranes by controlling the polyethylene segment content of EVOH.

As concluded above, the hydrophilicity of the membrane is derived from EVOH, and the increase in the blocked factor reduces the contact angle, but the decrease in hydroxyl increases the contact angle. The results show that the contact angle has a significant increasing trend, so it is obvious that the decrease in hydroxyl plays a major role.

## 4. Conclusions

In this study, a hydrophilic polypropylene hollow-fiber membrane was prepared by the dissolution-induced porous method. It was found that the filtration performance of the membrane was controlled by the content and the structure of EVOH. The increase in EVOH content decreased the embedded factor, increased the blocked factor and the hydrophilicity of the membrane, made the microfibrillation more pronounced, and increased the porosity. The increase in the polyethylene segment of EVOH increased both the embedded factor and blocked factor, decreased the hydrophilicity of the membrane, caused the microfibrillation to disappear, and decreased the porosity. The preparation of PP hollow fiber prepared by the DIP method should lay the foundation for the further development of the DIP method.

## Data Availability

Data is contained within the article.
